# Artificial intelligence algorithm for real-time detection and counting of *Trypanosoma cruzi* parasites using smartphone microscopy

**DOI:** 10.1371/journal.pntd.0012955

**Published:** 2026-05-07

**Authors:** Lin Lin, Ana Valeria Solano, Fabiola Gonzales, Mary Cruz Torrico, Daniel Illanes, Nuria Díez, David Bermejo-Peláez, Elena Dacal, Ramón Vallés-López, Lucia Pastor, Roberto Mancebo-Martín, María Jesús Ledesma-Carbayo, Miguel Luengo-Oroz, Jose M. Rubio, Maria Flores-Chavez

**Affiliations:** 1 Spotlab, Madrid, Spain; 2 Biomedical Image Technologies, ETSI Telecomunicación, Universidad Politécnica de Madrid, Madrid, Spain; 3 CIBER de Bioingeniería, Biomateriales y Nanomedicina, Instituto de Salud Carlos III, Madrid, Spain; 4 Facultad de Medicina, Universidad Mayor de San Simón, Cochabamba, Bolivia; 5 National Centre for Microbiology, Instituto de Salud Carlos III, Madrid, Spain; 6 Centro de Investigación Biomédica en Red de Enfermedades Infecciosas (CIBERINFEC) Instituto de Salud Carlos III, Madrid, Spain; 7 Fundación Mundo Sano, Madrid, Spain; Advanced Centre for Chronic and Rare Diseases, INDIA

## Abstract

Chagas disease affects 6–7 million people worldwide and causes approximately 12,000 deaths annually. Diagnostic methods vary by disease stage, with serological tests commonly used in the chronic phase, while microscopy and molecular techniques like PCR and LAMP are employed in the acute phase. While microscopy remains the most accessible tool in resource constrained settings, its effectiveness depends on skilled personnel, creating diagnostic bottlenecks. To overcome these limitations, we developed a portable, smartphone-integrated AI system for real-time *Trypanosoma cruzi* detection in microscopy images. The platform combines a 3D-printed microscope adapter which aligns the smartphone camera with the microscope ocular to digitize images, with telemedicine-enabled annotation workflows, and lightweight AI models (SSD-MobileNetV2, YOLOv8) deployed on smartphone for real-time analysis. Trained on a diverse dataset of human samples (478 images from 20 samples), including thick/thin blood smears and cerebrospinal fluid) and murine thin smears (570 images from 33 samples), the SSD-MobileNetV2 model achieved 86% precision, 87% recall, and 86.5% F1-score on human samples, demonstrating robust performance across variable imaging conditions. We additionally piloted a real-world experiment with the proposed system. Three thin blood smears were scanned by a user operating the smartphone-based system, with predictions generated in real time. Model outputs were benchmarked against expert annotations as the ground truth. At the object level, the algorithm achieved a precision of 67.1%, a recall of 96.4%, and an F1-score of 79.1%, showing high sensitivity under operational conditions with a configuration possibly suitable for screening. This system could enable rapid, accurate parasite detection in field settings without advanced infrastructure, addressing critical gaps in early diagnosis and monitoring. Its modular design allows adaptation to other pathogens and cellular structures, offering a scalable solution for neglected tropical disease diagnostics. By bridging AI innovation with microscopy, this approach holds promise for advancing equitable healthcare delivery in endemic regions and aligning with global health priorities.

## Author summary

Chagas disease is a life-threatening illness affecting millions, primarily in Latin America, where access to advanced laboratory equipment and trained specialists is limited. One method of diagnosis is microscopic examination of blood or cerebrospinal fluid samples, it provides immediate results without requiring complex facilities, but its effectiveness depends on the expertise of trained microscopists.

We developed a simple, low-cost tool that combines a smartphone, a light microscope, and artificial intelligence (AI) to assist in the diagnosis. By attaching a smartphone to a microscope with a 3D-printed adapter, users can analyze microscopy images with real-time AI assistance, detecting the parasites causing Chagas disease.

Our results show that this approach could be useful for regions with limited healthcare infrastructure. It reduces reliance on specialized training and expensive equipment, helping communities diagnose Chagas faster.

Beyond Chagas, this approach can be adapted to detect other diseases, offering a versatile tool for fighting neglected tropical illnesses. AI can be used to bridge knowledge gaps and support healthcare access. We hope this technology can empower frontline workers and contribute to global efforts to reduce the burden of these diseases on vulnerable populations.

## Introduction

Chagas disease, also known as American trypanosomiasis, is a life-threatening illness caused by infection with the protozoan parasite *Trypanosoma cruzi*. It affects about 6–7 million people worldwide and is endemic in 21 Latin American countries, leading to approximately 12,000 deaths every year [1]. The *T. cruzi* is mainly transmitted by contact with feces/urine of triatomine bugs, but can also be transmitted via consumption of contaminated food or beverages, congenital, transfusional, organ transplantation and laboratory accidents.

The disease presents two phases, during the initial acute phase, a high number of parasites circulates in the blood, but the symptoms are mild or absent, including fever, headache, enlarged lymph glands, etc. In particular situations, myocarditis and meningoencephalitis can be observed. The acute phase is followed by the chronicle phase, with the parasites hidden in the hearts and digestive muscle, causing cardiac, digestive, or neurological disorder, leading to death [2]. In immunosuppressive conditions such as HIV co-infection, parasitemia increases to levels similar to those in the acute phase, and may cross the blood-brain barrier, causing cerebral chagoma [3,4].

The diagnosis of Chagas disease can vary depending on the stage of the disease (acute or chronic) and the resources available. During the chronicle phase, the diagnosis is mainly performed using serological tests, including Enzyme-Linked Immunosorbent Assay (ELISA) and Indirect Immunofluorescence Assay (IFA) due to their ability to detect antibodies against *T. cruzi* [5,6]. In contrast, the acute phase is characterized by high parasitemia, enabling direct parasite detection through microscopy or molecular techniques, including PCR and loop-mediated isothermal amplification (LAMP) [7]. While molecular methods offer rapid and highly sensitive detection of the parasitaemia, their reliance on costly equipment, specialized training, and advanced laboratory infrastructure limits their utility in resource-constrained settings. Microscopy, on the other hand, remains a cornerstone of acute-phase diagnosis due to its ability to visualize *T. cruzi* trypomastigotes in fresh blood or cerebrospinal fluid smears (CSF) samples or stained smears. This method provides immediate results without requiring complex facilities, though its effectiveness depends on the expertise of trained microscopists to ensure high clinical sensitivity and specificity [7–9].

Microscopy is particularly valuable in regions where Chagas disease overlaps with other endemic infections, such as malaria. For example, in the Brazilian western Amazon—a region with high malaria prevalence and sporadic Chagas outbreaks linked to oral transmission via contaminated açaí juice—febrile illnesses are often presumptively attributed to *Plasmodium* infections [10]. However, microscopy enables the incidental identification of *T. cruzi* during routine blood smear analysis for malaria, as the trypomastigotes exhibit distinct morphological features distinguishable from malaria parasites [11]. This dual diagnostic capacity has promoted initiatives in the Brazilian Amazon to train microscopy personnel in recognizing both pathogens, enhancing early detection and treatment in areas with limited access to advanced diagnostic tools [11].

Recent advancements in artificial intelligence (AI) are poised to address key limitations of traditional microscopy while amplifying its strengths. AI-driven tools are increasingly being applied to medical image analysis [12,13] In addition to radiology, AI also holds great potential for microscopy image analysis, including pathology [14,15], hematology [16,17], and parasitology [18,19], such as malaria [20–22], Schistosoma [23–25], soil-transmitted helminthiasis [26–29] or filariasis [30]. For Chagas disease, AI models have shown promise in detecting *T. cruzi* trypomastigotes in blood smears with high accuracy. Researchers have employed various image processing techniques combined with AI to detect and quantify *T. cruzi* parasites. The algorithms developed for this purpose can be classified into three main categories: the first category involves traditional image processing methods to extract relevant features, which are then analyzed using machine learning algorithms; the second category relies on convolutional neural networks (CNNs) for automatic feature extraction from raw images, enabling the classification of images as infected or non-infected; and the third category utilizes CNN-based segmentation methods that not only determine infection status but also provide detailed information on the distribution of parasites within a sample [31–33].

In the first category, Soberanis et al. proposed an approach where the image is first segmented and then classified using the k-nearest neighbor algorithm [34]. Uc-Cetina et al. developed a parasite detection method using AdaBoost and Support Vector Machine (SVM), which achieved a sensitivity of 100% and a specificity of 93.25% [35]. Both studies used the same dataset, which consisted of blood samples from infected mice, digitized at 100x magnification, and cropped to 256x256 pixels. Another notable work within this category is by Morais et al., who introduced a system that combines graph-based segmentation with random forest classification. In this method, images acquired via mobile phones were segmented using a graph-based technique, with areas of interest cropped into 100x100 patches, which were then classified using a random forest algorithm. This system achieved a precision (positive predictive value) of 88.6% and a recall (sensitivity) of 90.5% [31].

The second category includes the work of Pereira et al., who proposed the first image classification algorithm using CNNs to identify whether a 224x224 pixel patch contains *T. cruzi* parasites. Their model, based on MobileNet and trained on 331 image patches with testing on 214 patches, achieved a precision of 91.8% and a recall of 50.5% [32]. Similarly, Jung et al. utilized CNN ResNet18 for classifying 224x224 pixel patch images, achieving a sensitivity of 70.6% and specificity of 65.1% [36].

In the third category, segmentation using CNNs has been a focal point. Ojeda et al. proposed a U-Net based approach for segmenting images of 512x512 pixels, which resulted in a precision of 63.04% and a recall of 87.02% [37]. In another study, Sanchez-Patiño et al. applied U-Net for the segmentation of *T. cruzi* parasites in histopathological images, achieving a binary accuracy of 98.19% [33].

Despite the promising advancements in applying AI and image processing techniques for diagnosing Chagas disease, several drawbacks and limitations persist in these works that must be addressed to improve their clinical utility and reliability. One significant limitation of those algorithms is the ability to execute the algorithm at the point of care in almost real time. For algorithms of the first and second category, they use small patches covering a small area of the sample preparation, for a single field of view, several patches are generated, leading to computational inefficiency. On the other hand, the segmentation algorithms usually have high computational complexity, posing a challenge on the deployment of the algorithm on resource-constrained settings.

Building upon the challenges of existing methods, in this work we present a comprehensive methodology for the development and validation of a lightweight AI algorithm for the detection of *T. cruzi* parasites. Our process covers the initial key stages of image digitization with smartphones coupled to a light microscope and data labeling via a telemedicine platform, which are detailed in previous work [29,30]. The main contribution of this paper centers on the development and optimization of the AI model for mobile hardware. We also performed an experiment piloting the resulting AI algorithm in a lab environment. Overall, this work establishes the foundation for a point-of-care solution that could be used for screening, early detection and monitoring of Chagas disease in endemic regions, and contributing to the World Health Organization’s targets for controlling Neglected Tropical Diseases [38].

## Materials and methods

### Ethical statement

Ethical approval was obtained from the Comité Bioética de la Facultad de Medicina (approved on 30/10/2020), Universidad Mayor de San Simón, Bolivia and from the Research Ethics Committee (REC) Instituto de Salud de Carlos III, Spain (CEI PI 74_2020).

### Overall workflow

**Fig 1:**
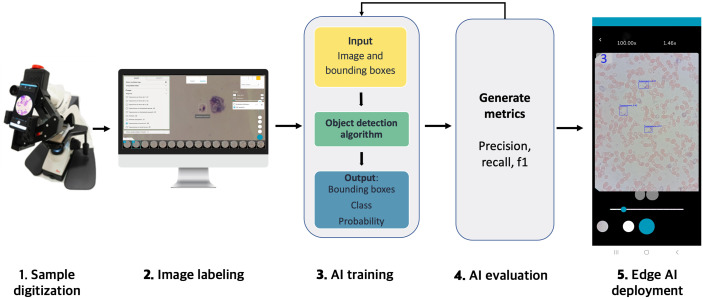
Simplified study design and workflow.

This study details a comprehensive workflow for developing a computer-assisted diagnostic tool, from sample digitization to on-device deployment (Fig 1). The workflow begins with sample collection and digitization using a smartphone based system. The digitization process, detailed previously [29,30], involves attaching the smartphone to the ocular of a conventional optical microscope via a 3D-printed adapter. A custom Android application enables users to digitize the microscopy field of view and upload the images to a telemedicine platform for expert annotation, placing a bounding box around each identified parasite, thereby creating the necessary training dataset. To enable parasite localization and quantification, we selected AI models for object detection as the core methodology. Accordingly, we trained and evaluated three distinct object detection models: SSD-MobileNetV2, YOLOv8-Small, and Faster R-CNN with a ResNet-50 backbone. Finally, the best-performing model was optimized for deployment on smartphones without requiring internet connectivity to assist clinicians in the diagnostic process in real time.

### Dataset

**Fig 2:**
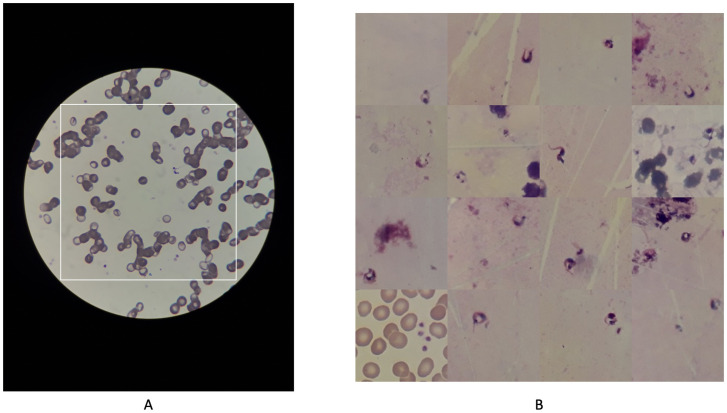
Data preprocessing. **(A)** Field of view image, digitized with smartphone. **(B)** Mosaic augmentation example.

Our dataset comprises two subsets: we created a new dataset (Laboratorios de Investigación Médica (LABIMED) dataset) consisting of human thick and thin blood smears and cerebrospinal fluid smears (CSF), and a publicly available dataset containing mice thin blood smears (Fig 2). Integrating these diverse samples exposed the model to greater clinical and experimental variability. This process was crucial for improving the model’s adaptability, as sample preparation, staining protocols, and imaging conditions often differ between laboratories. For instance, human thick blood smears retain multiple blood cell layers, whereas thin smears provide single-cell layers, altering parasite morphology and background noise. Similarly, staining methods affect color contrast and parasite visibility. Variations in imaging equipment - such as microscope resolution, lighting conditions, or camera - further compound these discrepancies.

A total of 36 smear preparations were collected from LABIMED, a reference laboratory for parasitology in Cochabamba, Bolivia, involving 9 positive subjects. Among these, 8 provided blood smear samples and 1 provided a cerebrospinal fluid (CSF) sample. All samples were stained with Giemsa. Specifically, 9 preparations from a single subject were CSF samples, 13 preparations from 4 subjects were thin blood smears, and 14 preparations from another 4 subjects were thick blood smears. The samples were digitized using a Samsung Galaxy J7 prime smartphone coupled to an Olympus CH30 microscope. Images were acquired through a 100x oil immersion objective with a numerical aperture of 1.25, creating final images of 4128x3096 pixels. Digitization was made using a 3D printed adapter coupled into the microscope, allowing the use of conventional microscopes to collect the images. Both positive and negative images were captured, resulting in 726 images.

The public dataset comprises 33 mice thin blood smear samples, totaling 675 images [31]. All images were Giemsa-stained and digitized using a smartphone camera coupled to an optical microscope with a 100x objective. Most images were captured at a resolution of 3,456 × 4,608 pixels, though a subset (2,448 × 3,264 pixels) reflects adjustments in camera settings during acquisition.

### Image labeling

For the development and evaluation of our object detection algorithm for *Trypanosoma cruzi*, accurate image labeling was crucial. We started by labeling the LABIMED dataset from scratch. Bounding boxes were placed around the identified parasite, indicating the location and the class of the parasite. In addition, some artifacts that look similar to the parasite were annotated. A total of 931 parasites were identified and annotated. Then, we trained a preliminary model to generate predictions on Morais *et al.* dataset, these predictions were subsequently reviewed and refined by a human annotator, who adjusted the bounding boxes to ensure they accurately aligned with the parasites and corrected any false positives. This iterative process led to the creation of 3,151 labeled objects.

The labeled dataset was carefully split into two independent sets at sample preparation level for LABIMED dataset. This approach ensured that all images from the same preparation belonged to the same dataset, whether used for training the AI model or validating its performance. The split was carried out after labeling the images to guarantee that each sample preparation type is presented in both the training and validation sets. The split was performed randomly, striving to achieve an 80%-20% split between the two sets. The result distribution is presented in Table 1.

**Table 1:** Dataset distribution for model training and validation.

	Training			Validation			Total		
Sample type	#sample	#image	#label	#sample	#image	#label	#sample	#image	#label
Human CSF	8	261	512	1	68	191	9	329	703
Human Blood thick	5	61	55	9	26	14	14	87	69
Human Blood thin	7	156	95	6	154	64	13	310	159
Mice Blood thin	–	570	2648	–	105	503	–	675	3151
Total	20	1048	3310	17	353	772	37	1401	4082

### Data preprocessing

**Fig 3:**
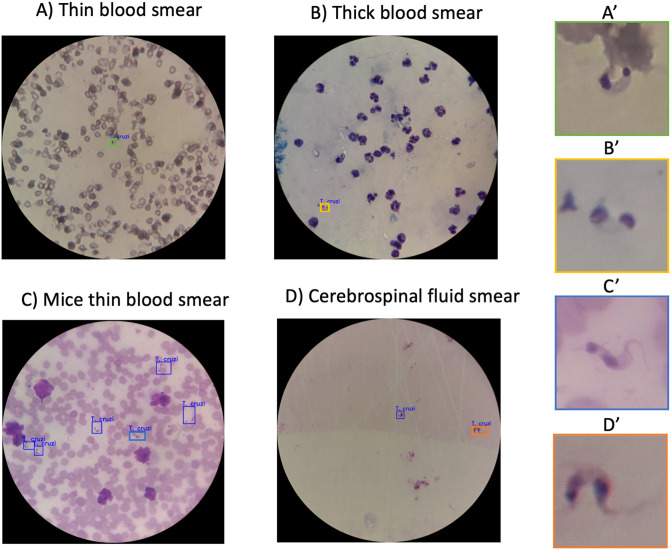
Microscopic images of blood and cerebrospinal fluid samples highlighting field of view and parasite visualization. The left panels display the full field of view for each sample type, while the right panels provide close-up views of parasites identified within the samples. Specifically, **A’** shows a parasite from the thin blood smear (Panel A), **B’** depicts a parasite from the thick blood smear (Panel B), **C’** presents a parasite from the mice thin blood smear (Panel C), and **D’** highlights a parasite from the cerebrospinal fluid smear (Panel D).

Given the alignment of the smartphone with the microscope eyepiece, the area captured by the mobile phone was limited to a circular region, as illustrated in Fig 3. As the parasite size is very small, typically occupying an area of only 100x100 pixels within an image that is 4128x3096 pixels in size, to exclude non-informative regions, such as the black areas outside the circular field of view, we opted to use square images. The size of the field of view is approximately 2800x2800 pixels, and the inner square is approximately 1900 × 1900 pixels. To increase the relative size of the parasites within the image, we selected square images that encompass the entire field of view, as represented by the white square in Fig 2. This approach ensured that the visualized area is maximized while focusing on the relevant regions for analysis.

In addition, to overcome the problem of imbalance foreground and background in object detection tasks, we manually increased the number of parasites by applying mosaic augmentation, which consisted of cropping a 160x160 pixel patches that contained at least one parasite, and blending 16 images to create a new image of 640 x 640 pixel. This augmentation was only applied for the training set.

### AI algorithm

For the automated analysis of microscopy images, our primary goal was not only to confirm the presence of *Trypanosoma cruzi* but also to locate and count each individual parasite. Simple image classification models, which only provide a label for an entire image (e.g., “parasite present”), are insufficient for this task as they do not provide location or quantity. Therefore, we chose an object detection approach. Object detection models are specifically designed to perform both classification (identifying an object as *T. cruzi*) and localization (placing a bounding box around each instance). This dual capability allows for the precise identification and enumeration of parasites within a given microscopic field, making it the ideal methodology for our study.

Object detection models can be divided into two groups: single stage detectors and two stage detectors. On the one hand, single stage detection algorithms are designed to make predictions within a single pass over the image which makes them faster but less accurate since they do not use a region proposal stage. On the other hand, two stage detection algorithms are more complex since they have a module that uses a CNN to propose regions of interest that are later refined in a second stage that performs the classification and localization. This process is usually more accurate but due to their nature, they are slower and require more computational resources.

In our work we focused on optimizing single-stage detectors to balance the need for speed and accuracy- as our goal was for the AI model to be run in a mid-range Android device in real time. This approach ensures that real-time detection capabilities are maintained. In particular, we evaluated two different algorithms that represent the two main approaches for single stage detectors: Single Shot Detection (SSD) and YOLO [39–41].

The SSD-MobileNetV2 model integrated the SSD framework with the lightweight MobileNetV2 architecture. SSD predicted bounding boxes and class probabilities directly from feature maps at different scales in a single pass, enabling efficient object detection. MobileNetV2, designed for mobile optimization, employs depthwise separable convolutions to minimize computational costs, making it ideal for deployment on resource-constrained devices like smartphones. This combination struck a balance between speed and accuracy. We initialized the model using pretrained weights from the COCO image dataset. For optimizing the loss function, we employed the RMSprop optimizer with an exponential decay learning rate, starting with a base learning rate of 0.008.

Conversely, YOLOv8 is one of the latest advancements in the YOLO series. It utilizes CSPDarknet53 as its backbone for feature extraction and incorporates a path aggregation network to enhance information flow across scales. YOLOv8 also introduces an anchor-free detection mechanism that predicts object centers directly, reducing the need for numerous box predictions and thus accelerating detection. The YOLOv8 family includes various variants, such as Nano, Small, Large, and Extra Large, each balancing accuracy and inference speed. For our application, we selected YOLOv8-Small for its optimal balance of these factors. The models were trained using the AdamW optimizer, configured with a learning rate of 0.002, a first momentum parameter of 0.9, and a second momentum parameter of 0.999. We also initialized the model using pretrained weights from the COCO image dataset for comparison purposes.

For both models, input size of 640x640 was used. During training, both models were subjected to on-the-fly data augmentation. These augmentations included random flips, rotations with a 50% probability, and random crops that maintained at least 80% of the original image area. We also implemented color jittering, randomly adjusting the image’s hue by a factor of ±0.015, saturation by a factor of 0.7, and value by a factor of 0.4. Additionally, early stopping with 10 epochs patience was employed to prevent overfitting, ensuring robust model performance.

For comparison purposes, we also trained a two-stage detector, Faster RCNN even though it is not suitable for smartphone deployment due to its higher computational demands. Faster R-CNN is a two-stage object detection model with high localization and classification accuracy. Our implementation utilized a ResNet-50 backbone to extract deep feature maps from the input images. Its first stage, the Region Proposal Network, identifies candidate object regions. These proposals are then passed to the second stage, where the model performs final classification and precise bounding box regression. This two-stage approach, while more computationally intensive than single-stage methods, allows the model to achieve a high degree of precision. For fair comparison, we also initialized this model using pretrained weights from the COCO dataset and used the RMSprop optimizer with a learning rate of 0.01 and momentum of 0.9.

We used two primary frameworks for model training to leverage the optimal tools for each architecture. The SSD-MobileNetV2 and Faster R-CNN models were trained using the TensorFlow Object Detection API. The YOLOv8-Small model was trained using the official Ultralytics framework, which provides state-of-the-art implementations specifically for the YOLO family of models. All training was conducted on the Amazon SageMaker platform, utilizing an NVIDIA A10G GPU with 16 GB of memory and a Thermal Design Power (TDP) of 150 W. The total training time for the project was approximately 42 hours, resulting in an estimated carbon footprint of 2.43 kg CO₂ eq.


$$\begin{array}{c} CO_{\text{2}}\;emissions(kg\;CO_{\text{2}}eq)=Power(kW)\;x\;duration(h)\;x\;Carbon\;intensity(kg\;CO_{\text{2}}/kWh) \end{array}$$

### AI model preparation for deployment

The trained AI model was exported to tflite format, with a size of approximately 11 megabytes for SSD Mobilenet and 45MB for YOLOv8 model. These models can be executed on Smartphones in real time. In addition, we created a customized Android application to execute the model. This app has the key requirement of working in real time without internet connectivity in order to be suitable for work in regions with intermittent or unavailable connectivity and/or electricity.

## Results

### Model performance on cross-validation and random split evaluations

Due to the limited sample size, we employed a two evaluation strategy to assess model performance. First, we performed five-fold cross-validation for each model to obtain a more reliable estimate while mitigating fold-to-fold variability. In parallel, we trained a version of each model using a random 80/20% split of the data to evaluate performance in a conventional training-testing scenario.

Following data preprocessing, the final training set comprised 7,249 augmented images with 66,557 annotated parasites, while the validation set, used to assess model generalizability, included 1,099 images containing 1,668 labeled targets. After training, the top-performing model checkpoint (selected based on validation performance) was used to analyze validation images, successfully identifying *T. cruzi* parasites in diverse microscopy samples as visualized in Fig 3.

To evaluate the performance of our models, we used precision, recall, and F1 score as metrics. For our evaluation, a detection was classified as a true positive if its confidence score exceeded the threshold and its Intersection over Union (IoU) with a ground truth box was greater than 0.3. A detection was classified as a false positive if it did not overlap with any ground truth box. Finally, a ground truth object was classified as a false negative if it was not detected by the algorithm. The threshold value was chosen to balance precision and recall, aiming to detect as many parasites as possible while limiting the number of false positives. If the priority is to ensure no parasites are missed, a lower threshold can be used to increase sensitivity. Conversely, to minimize false positives, a higher threshold can be applied. Precision, recall, and F1 score are then defined as follows:


$$\begin{array}{c} Precision=\frac{TP}{TP+FP} \end{array}$$

$$\begin{array}{c} Recall=\frac{TP}{TP+FN} \end{array}$$

$$\begin{array}{c} f\text{1}\;score=\text{2}x\frac{Precision\;*\;Recall}{Precision\;+\;Recall} \end{array}$$

Cross-validation across five folds revealed subtle differences in the performance of the three detection models. FasterRCNN-ResNet50 maintained consistently high F1-scores (85.8–94.5%), with slightly higher recall in fold 4 (93.6%) and lower in fold 2 (89.3%). SSD-Mobilenet v2 showed greater variability, particularly in recall (85.8–94.5%), achieving its highest F1-score in fold 4 (90.7%). YOLOv8-small generally had higher precision (91.3–92.8%) but lower recall (86.0–89.3%), resulting in moderate F1-scores (88.8–91.0%). Overall, while FasterRCNN-ResNet50 provided the most balanced and consistently high performance, the differences among the models were relatively small, suggesting all three architectures are competitive, with FasterRCNN offering a slight edge in reliability across folds (Table 2).

**Table 2:** Cross-validation performance summary (mean ± SD, %).

Model	Precision (%)	Recall (%)	F1-score (%)
FasterRCNN-ResNet50	91.2 ± 2.3	91.75 ± 1.8	91.4 ± 1.0
SSD-Mobilenet v2	88.5 ± 1.7	90.9 ± 3.1	89.2 ± 1.5
YOLOv8-small	92.0 ± 0.6	87.5 ± 1.2	89.7 ± 0.9

Building on these results, we further evaluated model performance using a random 80/20% data split to provide an additional, complementary assessment of model robustness (Table 3) SSD-MobileNetV2 demonstrated superior performance on human samples, achieving a precision of 86%, a recall of 87%, and an F1 score of 86.5%. In comparison, YOLOv8-Small recorded precision, recall, and F1 scores of 83.6%, 87%, and 80.5%, respectively. Conversely, YOLOv8-Small outperformed SSD-MobileNetV2 on mice samples, attaining precision, recall, and F1 scores of 93.7%, 91.3%, and 92.5%, while SSD-MobileNetV2 achieved 95.8%, 85%, and 90.1%, respectively. Notably, the two-stage detector Faster R-CNN exhibited higher performance than the single-stage detectors, with precision, recall, and F1 scores of 93.6%, 81.4%, and 87.1% on human samples, and 96.8%, 90.3%, and 93.5% on mice samples. These results complement the cross-validation findings, highlighting Faster R-CNN’s robustness and balanced performance across sample types.

**Table 3:** Model performance on human and mice samples, and the inference time measured on an Oppo Reno 6 smartphone.

	Human			Mice			Inference on smartphone (milliseconds)
Architecture	Precision	Recall	F1 score	Precision	Recall	F1 score	
SSD-Mobilenet v2	86	87	86.5	95.8	85	90.1	350
YOLOv8-Small	83.6	87	80.5	93.7	91.3	92.5	350
Faster R-CNN (ResNet 50)	93.6	81.4	**87.1**	96.8	90.3	**93.5**	–

The reported inference time measures the average duration to process a single image frame on our target hardware. This measurement begins when an image is received and ends when the final predictions are returned. It therefore encompasses all necessary steps: image pre-processing (e.g., resizing and normalization), the model prediction itself using the TensorFlow Lite interpreter, and post-processing to decode the output into usable bounding boxes.

When analyzing performance across different sample types, the models showed optimal results for mice with thin blood smears, achieving a precision of 95.8%, a recall of 85%, and an F1 score of 90.1%. In contrast, the performance on human thick blood smears was lower, with precision, recall, and F1 scores of 69.2%, 64.3%, and 66.7%, respectively. Performance for human thick smears drops compared to human CSF, thin, or mouse smears due to fewer training images and labels. These detailed results are presented in Table 4.

**Table 4:** Performance of SSD MobileNet v2 separated by sample type.

Model	Sample type	Precision	Recall	F1 score
SSD MobileNet v2	Human thick blood smear	69.2	64.3	66.7
	Human thin blood smear	91.8	87.5	89.6
	Human CSF smear	85.3	88.5	86.9
	Mice thin blood smear	95.8	85.0	90.1

### Pilot experiment of the AI-Augmented microscopy system in a laboratory environment

**Fig 4:**
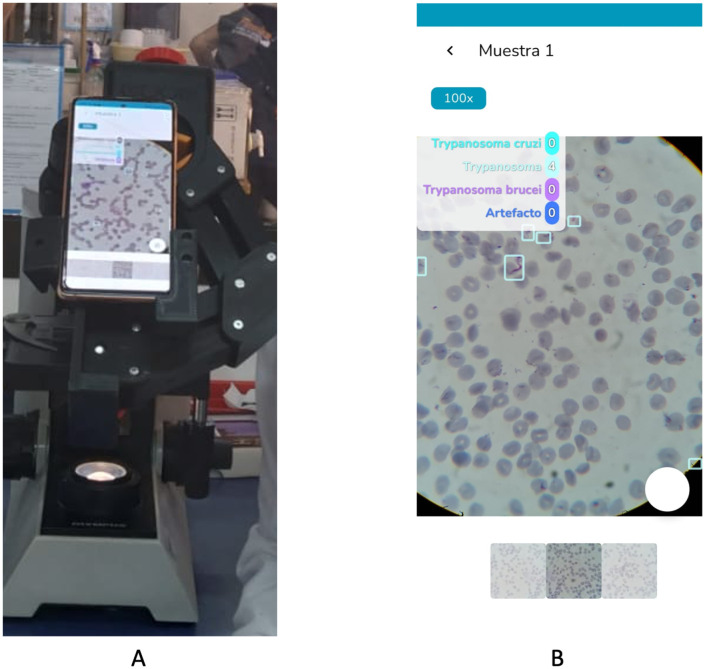
Operational deployment of real-time AI Algorithm. (A) Setup for image acquisition and real-time processing using a smartphone coupled to a light microscope. (B) Screenshot illustrating the real-time AI detection of parasites on the smartphone interface.

To complement the controlled cross-validation and random-split experiments, we further assessed the AI algorithm in an experiment in real-world conditions using independently collected blood smears and a smartphone-based scanning workflow (Fig 4). This approach allowed us to assess how the models perform outside of curated datasets in a lab environment capturing practical challenges including user interaction. By benchmarking the algorithm against expert annotations in a real-time scanning scenario, we were able to directly evaluate its robustness in conditions that closely mimic field deployment.

Three independent thin blood smears were used to assess the AI algorithm in real time in a lab environment. The evaluation protocol required a user to scan the smears using a smartphone running the algorithm in real-time. Images were saved under two conditions: (1) when the AI detected a potential parasite (true or false positive) and (2) when the user manually identified a parasite missed by the AI (false negative).

This approach of real-time AI augmented microscopy recorded the key fields of view, A single 15 × 15 cm scan at 100 × magnification can generate over 500,000 fields of view, making recording of every frame impractical unless a mechanical scanner is used. Consequently, only images containing potential findings were saved, while correctly identified negative fields (true negatives) were not recorded. All captured images and corresponding detections were then uploaded to the telemedicine platform for independent review by two experts: Expert A (senior) and Expert B (junior).

### Performance results

Inter-rater reliability between the two experts was strong, with a Cohen’s Kappa score of 0.868. This high agreement established confidence in the annotated ground truth, against which the AI algorithm was benchmarked (Expert A designated as the ground truth).

At the object level, the algorithm achieved a precision of 67.1%, a recall of 96.4, resulting in an F1-score of 79.1% (Fig 2). This performance profile suggests the system configuration is well-suited for screening applications.

This real-time evaluation showed the system’s operational practicality and the value of human oversight. The algorithm generated an average of seven false positive detections per scanned sample, a manageable rate of false alarms that can be efficiently reviewed and dismissed by the human user. The human operator also recovered the 3.6% of parasites missed by the algorithm detections among the recorded fields of view. This finding highlights how the human operator effectively leveraged the AI’s help with detections, while compensating for the AI’s misses, minimizing the likelihood of a critical error and affirming the value of the human-in-the-loop approach.

## Discussion and conclusion

In this work, we presented a comprehensive methodology for the development and validation of a lightweight AI algorithm for the automated detection of *T. cruzi* parasites, demonstrating its feasibility for deployment on AI-augmented smartphone microscopy.

Laboratory diagnosis plays a crucial role in the control and monitoring of diseases. In the case of Chagas disease, the *T. cruzi* parasites detection in microscopic images remains a widely used method for acute phase diagnosis. Within endemic areas, in specialized and clinical laboratories, during the manual leukocyte count in blood smears or *Plasmodium* thick blood smear examination, *T. cruzi* trypomastigotes could be visualized. In experimental settings, such as drug discovery, automated detection of *T. cruzi* in microscopy images is essential for efficiently assessing treatment efficacy. Mice models are commonly used to evaluate potential drugs by monitoring parasitemia levels before and after treatment. Manual counting of parasites is time-consuming and prone to human error, which can introduce variability in results. Automated detection streamlines this process, providing rapid, consistent, and objective measurements of parasite burden. This enables researchers to accurately track disease progression and therapeutic response, ultimately facilitating the development of more effective treatments for Chagas disease [42–44]. Although various studies have explored automating parasite detection, achieving promising results but still facing certain limitations. Many algorithms operate on small image patches, cropping field-of-view images into numerous small areas, which increases computational time [31,32,35,36]. Alternatively, segmentation algorithms such as U-Net have been employed, offering detailed pixel-level information about the shape and size of parasites [33,37]. While segmentation provides precise measurements, it requires extensive pixel-level labeling, which is more time-consuming than bounding box annotations. Moreover, in the experimental contexts, the primary goal is to count the number of parasites rather than to determine their exact shape. Therefore, object detection, which focuses on identifying and counting objects within an image, could be more suitable for these purposes. In the majority of previous studies, the use of mice samples without including human samples, introducing possible bias.

In response to the limitations observed in existing methods, our study introduces an object detection approach specifically designed for Chagas disease diagnostics. We utilize advanced single-stage detectors, namely SSD-MobileNetV2 and YOLOv8, to enhance the detection process for deployment on smartphones. Our approach benefits from a comprehensive dataset that includes various sample types and incorporates robust data augmentation techniques, ensuring effective generalization across diverse imaging conditions. Our approach leverages a diverse dataset and robust data augmentation to ensure effective generalization across imaging conditions. Both SSD-MobileNetV2 and YOLOv8-Small demonstrated strong performance, achieving metrics consistently above 80% for all sample types, with SSD-MobileNetV2 excelling in human samples. The SSD-MobileNetV2 model’s inference time is approximately 350 milliseconds on a smartphone, underscoring its efficiency and suitability for real-time mobile diagnostics. By enabling real-time inference, our approach significantly enhances the practicality of AI-assisted Chagas disease diagnosis in field settings, where internet connectivity and computational resources are limited. Immediate and automated parasite detection reduces reliance on trained experts, minimizes diagnostic delays, and improves accessibility to timely diagnosis.

Even though Faster R-CNN is not suitable for deployment on smartphones due to its computational demands, the model can still be effectively utilized in a cloud-based environment. This allows for its integration into a human-in-the-loop labeling process, where it can assist in automating the initial labeling of data. By leveraging the model’s high accuracy, this approach can significantly reduce the time and effort required for manual labeling, while still allowing human oversight to ensure the quality of the annotations.

We piloted the proposed system in a prospective experiment in a lab environment using independent blood smears. The algorithm achieved high sensitivity, successfully detecting the majority of parasite instances, suggesting how it could work as a screening system or as an initial step that can be quickly refined by the human specialist. The results indicate that the models’ strong performance in cross-validation and random-split experiments can translate effectively to field-like conditions. This experiment reinforces the idea that AI-assisted detection can support rapid and reliable diagnostics directly at the point of care, reinforcing its potential to improve accessibility and reduce diagnostic delays in resource-limited settings.

The primary limitation of our study is the sample size, particularly for human thick blood smears, where only 87 images with 69 labeled instances were available. This limited dataset likely contributed to the suboptimal performance for this sample type. We hypothesize this is due to the differences in parasite appearance and background characteristics between thick and thin smears, which affect the model’s ability to generalize across sample types. We believe that increasing the number of training samples could significantly enhance model performance, as evidenced by the strong results achieved with human thin blood smears, human CSF smears, and mice thin blood smears, where precision, recall, and F1 scores all exceeded 80%. Future work should focus on expanding the dataset, particularly for thick blood smears.

Additionally, microscopy has the potential to identify a wide range of parasites and cellular structures within the same image. Therefore, the AI should not be limited to detecting *T. cruzi* alone but should aim to function as a universal detector capable of identifying all discernible structures, such as malaria parasites, leishmania amastigotes, and cells, akin to a human microscopist. Furthermore, as multi-modal AI technology advances, these types of platforms have the potential to incorporate additional data sources, such as clinical and genetic data, a new generation of AI-driven diagnostics [45].

In conclusion, the proposed system offers real-time assistance in the diagnosis of Chagas disease by transforming a conventional light microscope into an intelligent point-of-care device. Our integrated telemedicine platform facilitates image storage and remote consultations while streamlining the process of rapid image labeling and AI model development. Overall, new advancements in AI in medicine have the potential to provide accessible knowledge resulting in diagnostics tools for a wide range of infectious diseases, enhancing diagnostic accuracy and supporting global efforts to achieve the WHO’s targets for combating NTDs [38].

## Supporting information

S1 File Ground truth bounding boxes (S1_Ground_truth_bboxes.csv). This dataset contains the manually annotated reference coordinates used as the gold standard for evaluating object detection accuracy across all test images.
(CSV)

S2 File SSD model predictions (S2_Predictions_ssd.csv). Tabulated detection results generated by the Single Shot MultiBox Detector (SSD) architecture, including predicted bounding box coordinates, class labels, and associated confidence scores.
(CSV)

S3 File YOLOv8s model predictions (S3_Predictions_yolov8s.csv). Tabulated detection results generated by the YOLOv8 small (YOLOv8s) architecture, used to assess the real-time performance and precision of the model.
(CSV)

S4 File Faster R-CNN model predictions (S4_Predictions_fasterRCNN.csv). Tabulated detection results generated by the Faster R-CNN architecture, representing the two-stage detector’s output for performance comparison.
(CSV)
